# Conditioned Medium from Malignant Breast Cancer Cells Induces an EMT-Like Phenotype and an Altered *N*-Glycan Profile in Normal Epithelial MCF10A Cells

**DOI:** 10.3390/ijms18081528

**Published:** 2017-08-01

**Authors:** Jia Guo, Changmei Liu, Xiaoman Zhou, Xiaoqiang Xu, Linhong Deng, Xiang Li, Feng Guan

**Affiliations:** 1 Institute of Biomedical Engineering and Health Sciences, Changzhou University, Changzhou 213164, China; guojia@jiangnan.edu.cn (J.G.); dlh@cczu.edu.cn (L.D.); 2 Key Laboratory of Carbohydrate Chemistry and Biotechnology, Ministry of Education, School of Biotechnology, Jiangnan University, Wuxi 214122, China; cmliu@jiangnan.edu.cn (C.L.); zxm.jm@msn.cn (X.Z); 1050312215@vip.jiangnan.edu.cn (X.X.); 3 School of Life Science, Northwest University, Shaanxi 710069, China

**Keywords:** conditioned medium, EMT, *N*-glycan, breast cancer, cell migration

## Abstract

Epithelial-mesenchymal transition (EMT) is a key process in cancer development and progression. Communication (crosstalk) between cancer cells and normal (nonmalignant) cells may facilitate cancer progression. Conditioned medium (CM) obtained from cultured cancer cells contains secreted factors capable of affecting phenotypes and the behaviors of normal cells. In this study, a culture of normal breast epithelial MCF10A cells with CM from malignant breast cancer cells (termed 231-CM and 453-CM) resulted in an alteration of morphology. CM-treated MCF10A, in comparison with control cells, showed a reduced expression of the epithelial marker E-cadherin, increased expression of the mesenchymal markers fibronectin, vimentin, *N*-cadherin, and TWIST1, meanwhile cell proliferation and migration were enhanced while cell apoptosis was decreased. *N*-glycan profiles of 231-CM-treated and control MCF10A cells were compared by MALDI-TOF/TOF-MS (Matrix-Assisted Laser Desorption/ Ionization Time of Flight Mass Spectrometry) and a lectin microarray analysis. The treated cells showed lower levels of high-mannose-type *N*-glycan structures, and higher levels of complex-type and hybrid-type structures. Altered *N*-glycan profiles were also detected in 453-CM-treated and non-treated MCF10A cells by MALDI-TOF/TOF-MS, and we found that the expression of five fucosylated *N*-glycan structures (*m*/*z* 1406.663, 1590.471, 1668.782, 2421.141, and 2988.342) and one high-mannose structure *m*/*z* 1743.722 have the same pattern as 231-CM-treated MCF10A cells. Our findings, taken together, show that CM derived from breast cancer cells induced an EMT-like process in normal epithelial cells and altered their *N*-glycan profile.

## 1. Introduction

Epithelial-mesenchymal transition (EMT) is an important process in disease development, particularly in tumor metastasis [1]. During the EMT process, cells: (i) undergo a morphological change from epithelial to dispersed mesenchymal; (ii) display a reduced expression of epithelial cell marker molecules such as E-cadherin (E-cad), and an increased expression of mesenchymal cell marker molecules such as fibronectin (FN), *N*-cadherin (*N*-cad), and vimentin; and (iii) acquire malignant properties such as increased migration and invasiveness [1,2].

Glycosylation, catalyzed by specific glycosyltransferases and glycosidases, plays key roles in a variety of cell physiological processes, including protein folding, cell-cell adhesion, host-pathogen interactions, and cell signaling [3]. Aberrant glycosylation is often associated with malignant transformation and tumor progression. Altered *N*-glycan expression is accompanied by EMT [4,5,6]. In a 2014 study of normal mouse mammary gland epithelial (NMuMG) cells, we used a systematic glycomic analysis to demonstrate that the aberrant *N*-glycosylation of transforming growth factor β (TGF-β)-induced EMT enhanced the expression of high-mannose-type *N*-glycan structures, and reduced the expression of bisecting GlcNAc (*N*-acetylglucosamine) and fucose (Fuc) structures [7]. Our 2014 study of normal bladder epithelial HCV29 cells revealed a striking increase of high-mannose-type glycan structures and a reduction of biantennary *N*-glycan structures during EMT [8].

Cancer progression is affected by the communication (crosstalk) between cancer cells and normal cells. A 2010 study indicated that certain cancer cells are able to transfer DNA, microRNA, mRNA, lipids, and/or proteins associated with invasiveness to adjacent or remote normal cells, and thereby induce an EMT-like phenotype [9]. The mechanism of such transfer was unclear.

In the present study, we generated conditioned medium (CM) from MDA-MB-231 and MDA-MB-453 malignant breast cancer cells, and used it for the culture of MCF10A non-tumorigenic mammary epithelial cells. As a positive control, MCF10A cells were treated with TGF-β, an EMT inducer [10]. Cell proliferation, migration, apoptosis, and the expression of EMT markers were assayed in the CM-cultured MCF10A cells, and possible alteration of the *N*-glycan profile was investigated by MALDI-TOF/TOF-MS and a lectin microarray analysis. Our findings demonstrated that CM derived from breast cancer cells induced an EMT-like process in normal epithelial cells and altered their *N*-glycan profile.

## 2. Results

### 2.1. CM Induces an Increased Expression of EMT Markers in MCF10A Cells

**Figure 1:**
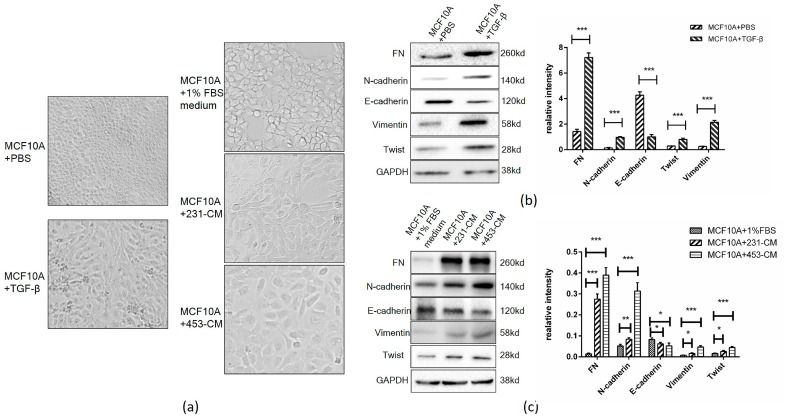
Cell morphology and expression of epithelial-mesenchymal transition (EMT) markers in MCF10A cells following TGF-β and conditioned medium (CM) treatment. (**a**) The morphological changes. The cells were cultured in 6-well plates and treated with CM for 24 h (**Right**). The cells cultured in DMEM (dulbecco’s modified eagle medium) with 1% fetal bovine serum (FBS) were used as control. The morphological changes of TGF-β-treated cells (**Left**) were compared with those of the CM-treated cells. The photos were taken under phase-contrast microscopy (40×); (**b**) Comparative expression of EMT markers in TGF-β-treated vs. PBS (phosphate buffered saline)-treated cells. Protein (10 μg/well) was subjected to SDS-PAGE, and the expression of EMT markers *N*-cad, E-cad, FN, Vimentin, and TWIST1 was analyzed by Western blotting (GAPDH (reduced glyceraldehyde-phosphate dehydrogenase) as control). Histograms was used to quantify the Western blot data. *** *p* < 0.001; (**c**) Comparative expression of EMT markers in CM-treated vs. DMEM/1% FBS-cultured cells. Western blotting was performed as above. Histograms were used to quantify the Western blot data. * *p* < 0.05, ** *p* < 0.01, *** *p* < 0.001.

During EMT, cells switch from an epithelial to a mesenchymal morphology. CM derived from certain cancer cells promotes cancer development in normal cells because it contains secreted factors [11,12,13,14]. In the present study, a culture of mammary epithelial MCF10A cells with CM from breast cancer cells MDA-MB-231 and MDA-MB-453 (termed 231-CM and 453-CM) resulted in a morphological change from epithelial-shaped to spindle-shaped, similarly to TGF-β-induced EMT (Figure 1a).

The EMT process is typically associated with a reduction of the epithelial protein marker *E*-cad, and increased levels of mesenchymal protein markers (*N*-cad, FN, Vimentin, TWIST1, and others). The TGF-β-treated MCF10A cells displayed downregulation of E-cad and upregulation of FN, *N*-cad, Vimentin, and TWIST1 (Figure 1b). Both 231-CM and 453-CM-treated MCF10A cells displayed this same pattern (Figure 1c), indicating that malignant cancer cell-CM induces an EMT-like process in these cells.

### 2.2. CM Promotes Proliferation and Migration But Inhibits the Apoptosis of MCF10A Cells

**Figure 2:**
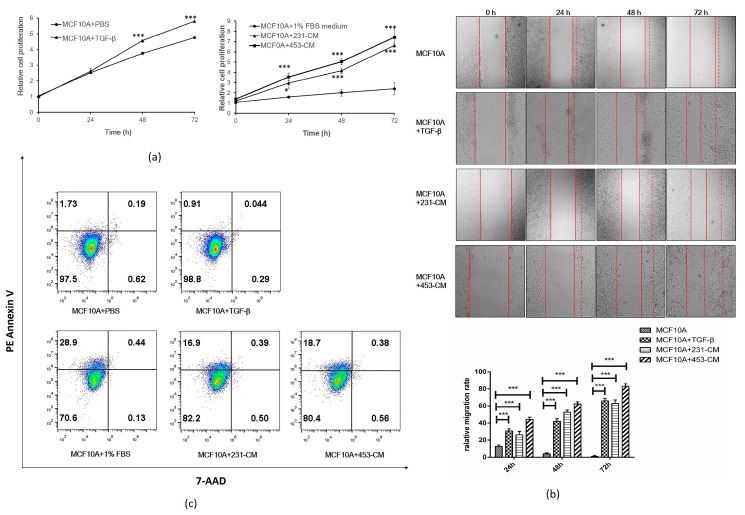
Proliferation and migration of TGF-β-treated and CM-treated MCF10A cells. (**a**) MTT assay of cell proliferation. Cells were seeded in 96-well plates and treated with 231-CM and 453-CM for 24, 48, or 72 h. DMEM/1% FBS-cultured cells were used as control. Proliferation was compared for TGF-β-treated vs. CM-treated cells. The results shown are mean ± standard error of measurement (SEM) from three independent experiments. * *p* < 0.05 ,*** *p* < 0.001; (**b**) Wound assay of cell migration. The cells were cultured in 24-well plates to high confluence (>80%), scratched with a 200-μL pipette tip at the marked position, washed twice with PBS, cultured in fresh medium with 1% FBS, and treated with TGF-β or 231-CM or 453-CM for 24, 48, or 72 h. Nontreated cells were used as control. Wounds were photographed at the marked position at the above times under phase-contrast microscopy (10×). Histograms was used to quantify the wound assay data. *** *p* < 0.001; (**c**) Cell apoptosis analysis. The cells were cultured in 6-well plates and treated with TGF-β or CM for 24 h. A PE (Phycoerythrin) Annexin V Apoptosis Detection Kit I was used to stain the apoptosis cells. Cells stained with PE Annexin V were identified as the early apoptotic cells (7-AAD (7-Aminoactinomycin D) negative, PE Annexin V positive), and cells that were in late apoptosis or were already dead were both PE Annexin V and 7-AAD positive.

MTT (3-(4,5-dimethyl-2-thiazolyl)-2,5-diphenyl-2-*H*-tetrazolium bromide) assay revealed the significantly enhanced proliferation of TGF-β-treated and CM-treated MCF10A at 24, 48, and 72 h (Figure 2a). The wound assay showed increased migration at various time points in both TGF-β-treated and CM-treated MCF10A cells (Figure 2b). A flow cytometric analysis exhibited decreased early and late apoptotic ratios of TGF-β- and CM-treated cells compared with the control (Figure 2c). These findings suggest again that a culture of MCF10A with CM results in an acquisition of EMT-like malignant behaviors.

### 2.3. N-Glycan Profiling of CM-Treated MCF10A Cells by MALDI-TOF/TOF-MS

*N*-Glycosylation is involved in cell recognition, receptor activation, cell signaling, and cell adhesion. Altered *N*-glycan expression is observed in many types of tumor cells [15]. In view of our findings above that culture with CM derived from malignant cancer cells induced an EMT-like phenotype in MCF10A cells, we used MALDI-TOF/TOF-MS to compare the total *N*-glycan expression in CM-treated vs. non-treated (control) MCF10A cells. *N*-glycan peaks in mass spectra were distinguished using a signal-to-noise ratio >5 as the criterion, and *N*-glycans were annotated using the GlycoWorkbench 2.1 program (http://code.google.com/p/glycoworkbench/).

**Figure 3:**
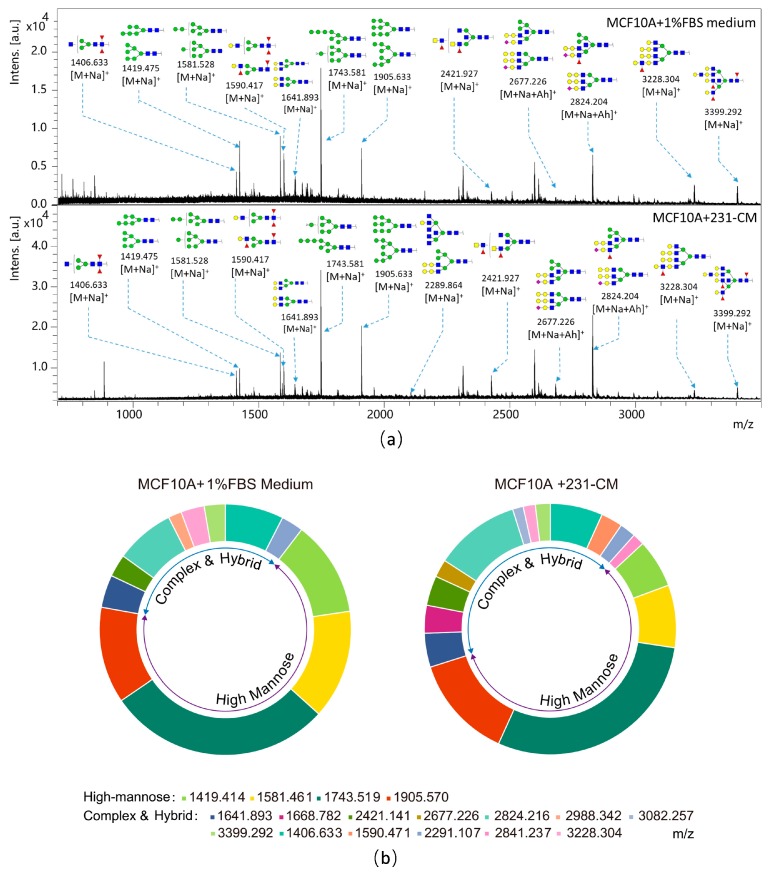
The alteration of *N*-glycan profiles in MCF10A cells was induced by 231-CM. (**a**) MALDI-TOF/TOF-MS spectra of *N*-glycans. The cells were cultured in a 10 cm dish, and the *N*-glycans were separated and desalted as described in Materials and Methods. Lyophilized *N*-glycans were dissolved in methanol/H_2_O (MW), and an aliquot of mixture with DHB (2,5-dihydroxybenzoic acid) solution was spotted on an MTP (maldi target plate) AnchorChip sample target and air-dried. MALDI-TOF/TOF-MS was performed in positive-ion mode. The experiments were performed in triplicate, and representative *N*-glycan spectra are shown. Peaks (signal-to-noise ratio >5) were selected for a relative proportion analysis. Detailed structures were analyzed using the GlycoWorkbench program. Proposed structures are indicated by *m*/*z* value. **Top**: 231-CM-treated cells. **Bottom**: DMEM/1% FBS-incubated cells; (**b**) Relative variation of various types of *N*-glycans in 231-CM-treated cells. Different colors represent *m*/*z* values as indicated.

Twelve distinct *m*/*z* *N*-glycan structures were found in the control MCF10A cells, and sixteen in 231-CM-treated MCF10A cells. Eleven structures were common to the control and 231-CM-treated cells, but with differing intensities. One structure was unique to the control cells, and five structures were unique to the 231-CM-treated cells (Table 1; Figure 3a). In the 453-CM-treated MCF10A cells, we also detected 26 *N*-glycan structures in both the control and 453-CM-treated cells with different intensities (Table S1). The expression patterns of five fucosylated *N*-glycan structures (*m*/*z* 1406.663, 1590.471, 1668.782, 2421.141, and 2988.342) and one high-mannose structure *m*/*z* 1743.722, have the same pattern as 231-CM-treated MCF10A cells.

The relative variations of the major types of *N*-glycans detected in the control and 231-CM-treated MCF10A cells are summarized in Table 2 and Figure 3b. The proportion of high-mannose-type *N*-glycans was marginally lower in the 231-CM-treated cells (56.99%) than in the control cells (67.50%), whereas the proportions of complex-type, hybrid-type, and multi-antennary (bi-, tri-, and tetra-antennary) *N*-glycan structures were higher in the 231-CM-treated cells. The levels of bisecting GlcNAc structures (45.46% vs. 16.32%) and of fucosylated *N*-glycan structures (45.91% vs. 17.71%) were significantly higher in the 231-CM-treated cells than in the control cells. In the 453-CM-treated cells, we detected the same variation patterns of bi-antennary *N*-glycan structures (7.7% vs. 9.5%) and fucosylated *N*-glycan (19.1% vs. 21.7%) compared with the 231-CM-treated cells (Table S2).

### 2.4. Lectin Microarray Analysis Reveals Altered Glycopattern in 231-CM-Treated MCF10A Cells

**Figure 4:**
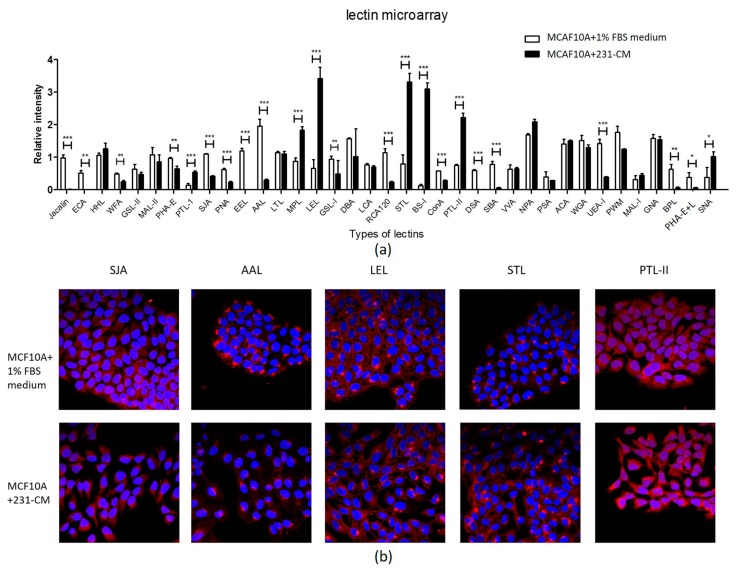
Lectin microarray analysis of glycan variation in 231-CM-treated MCF10A. (**a**) Variation of expression of the glycans recognized by 37 lectins as indicated. * *p* < 0.05; ** *p* < 0.01; *** *p* < 0.001; (**b**) Lectin staining analysis of altered glycan expression. Five lectins (SJA, AAL, LEL, STL, and PTL-II) were applied, and lectin staining was performed as described in Materials and Methods. Signals are shown from a merge image of Cy3-conjugated lectins and DAPI (4′,6-diamidino-2-phenylindole) staining of the nuclei in control (**top**) and 231-CM-treated (**bottom**) cells (magnification 60×).

A high-throughput analysis of the glycan structures was performed using lectin microarrays. Proteins from the control and 231-CM-treated MCF10A cells were incubated with 37 different lectins. Differences were considered significant if fold change of fluorescent intensity was >1.5 or <0.67. The 231-CM-treated MCF10A cells displayed higher binding affinity for seven lectins (BS-I, LEL, STL, PTL-I, PTL-II, SNA, and MPL) and lower binding affinity for 16 lectins (PHA-E, WFA, GSL-I, ConA, SJA, PNA, UEA-I, RCA120, PHA-E+L, AAL, BPL, SBA, DSA, jacalin, ECA, and EEL) (Figure 4a; Table 3).

The lectin microarray results were confirmed by a lectin staining analysis. The 231-CM-treated MCF10A cells showed significantly increased binding signals with LEL (*Lycopersicon esculentum* (tomato) lectin; recognizes poly-LacNAc and (GlcNAc)n structures), STL (*Solanum tuberosum* (potato) lectin; recognizes GlcNAc oligomer structure), and PTL-II (*Psophocarpus tetragonolobus* lectin II; recognizes Gal structure), and decreased binding signals with SJA (*Sophora japonica* agglutinin; recognizes terminal GalNAc and Gal structures) and AAL (*Aleuria aurantia* lectin; recognizes Fuc structure) (Figure 4b; Table 3). These findings were consistent with those from the lectin microarray analysis.

## 3. Discussion

Intercellular communication is essential for normal physiological cellular events. Cells deliver information by secreting factors such as proteins, DNA, RNA, and lipids. Conditioned medium (CM) contains such secreted factors, and may play key roles during cell-to-cell communication. A 2014 study suggested that secreted factors in stem cell-derived CM promote tissue repair under various conditions, and are potentially useful in regenerative medicine [16]. CM derived from a liver cell line enhanced the myofibril organization in primary rat cardiomyocytes, through factors [17]. In the present study, CM from malignant breast cancer cells produced an EMT-like process when used in a culture of MCF10A normal breast cells (Figure 1 and Figure 2).

Crosstalk between malignant cancer cells and normal stromal and parenchymal cells promotes tumor growth, angiogenesis, and metastasis through various secreted factors and their corresponding receptors [18]. CM from bone marrow-derived, CD271-expressing stromal cells enhanced the proliferation and motility of gastric cancer cells [12]. The chemotaxis of bone marrow-derived mesenchymal stromal cells via soluble signaling factors was induced by 231-CM [11]. CM from co-cultured stromal fibroblasts/head and neck squamous cell carcinoma (HNSCC) induced an EMT-like phenotype and decreased sensitivity to CDDP (Compound Danshen Dripping Pills) treatment in HNSCC cells [14]. In the present study, a culture of MCF10A cells with malignant breast cancer-CM induced changes similar to those observed in TGF-β-induced EMT.

EMT is an essential step in the development of solid tumor cells. During the EMT process, cells lose the expression of epithelial cell markers but acquire the expression of mesenchymal markers [19]. TGF-β is a common inducer of EMT, and it can activate the TGF-β/Smads signal pathway which regulates the expression of several transcriptional factors, e.g., Snail, TWIST1, Zeb1, and Slug, to start an EMT process [1]. Other than that, several signal factors such as Wnt, fibroblast growth factor (FGF) and epidermal growth factor (EGF) have been shown to participate in EMT [20].

Glycosylation widely exists in mammalian cells, and plays an important role in cell adhesion, motility, and cellular signaling events [21]. Many studies have demonstrated the involvement of aberrant glycosylation in EMT. The core α-1,6-fucose structure, which is catalyzed by fucosyltransferase-8 (FUT8) in mammals, has been reported to be upregulated in tumor progress [22,23], and participates in the regulation of the EGFR (Epidermal Growth Factor Receptor) signal pathway or in the regulation of the function of immunoglobulin [24]. An increased expression of sialic acids, which attach to the terminal of *N*-glycans and influence cell adhesion, has been considered as an important modification of many carcinomas [25]. The bisected GlcNAc structure formed by *N*-acetylglucosaminyltransferase III (GnT-III) has been reported to be downregulated during a TGF-β-induced EMT process [26], whereas the β1-6 branching of *N*-glycan structures catalyzed by *N*-acetylglucosaminyltransferase V (GnT-V) are increased in malignant cancers and EMT transition [27,28]. Altered glycan profiles were detected in hepatocellular carcinoma HUH7 cells during hepatocyte growth factor (HGF)-induced EMT by a lectin microarray [5]. The TGF-β treatment of HCV29 induces EMT and the alteration of the *N*-glycan expression profile [8]. An altered *N*-glycan profile was also associated with EMT in NMuMG [7]. In the present study, we focused on the influences of cell-cell communication on the synthesis of *N*-glycans, and found that the *N*-glycan profile of 231- and 453-CM-treated MCF10A cells differed from that of control cells. A MALDI-TOF/TOF-MS analysis revealed increases of complex-type and hybrid-type *N*-glycan structures, and a decrease of high-mannose-type structures in 231-CM-treated cells (Table 1 and Table 2; Figure 3), whereas increased expressions of bi-antennary *N*-glycan structures and fucosylated *N*-glycan were also detected in 453-CM-treated cells (Tables S1 and S2). The altered fucosylation has been reported in breast cancer, ovarian cancer, and many other cancers, and is associated with malignant cell behaviors [29]. Fucosylated glycoproteins such as AFP (α-fetoprotein) and CA 19-9, are used as tumor biomarkers of pancreatic and liver cancers, and new fucosylated glycoproteins have been found to serve as markers for small cell lung cancer [30]. Additionally, our results indicated that the fucosylation expression in normal cells could be affected by malignant breast cancer cells via cell-cell communications. Lectin microarray and lectin staining analyses revealed changes of various *N*-glycan structures in 231-CM-treated cells. The fluorescence intensities were increased for 7 lectins and reduced for 16 lectins in 231-CM-treated cells (Table 3).

Similarly to our findings, STL (recognizes oligomers of the GlcNAc structure) was reported to be upregulated in colon cancer tissues [31]. We found that STL and LEL (recognizes poly-LacNAc and (GlcNAc)_n_ structures) were upregulated in NMuMG during TGF-β-induced EMT [7]. An increase of the poly-LacNAc structure recognized by LEL was typically observed on β1-6 branches, which are synthesized by GnT-V and known to be enhanced in many types of cancers [32]. α2-6linked sialic acid (recognized by SNA) was upregulated following 231-CM treatment; this structure was more highly expressed in ovarian cancer cells relative to normal cells [33]. In contrast, bisecting GlcNAc (recognized by PHA-E) appears to act as a suppressor of many types of cancer [34,35]. This structure was reduced in the 231-CM-treated MCF10A cells in the present study, according to the lectin microarray analysis. All of the above findings indicate that the CM derived from malignant cells causes the alteration of *N*-glycan profiles of normal cells, and promotes cancer progression.

Many studies during the past two decades have demonstrated the diverse roles of cell-to-cell communication in the promotion of tumor metastasis. Our present findings show that CM derived from malignant breast cancer cells induces an EMT-like phenotype in normal breast epithelial cells and causes global changes in *N*-glycans. Follow-up studies are underway that focus on alterations of these glycans and related genes at the molecular level, the glycoproteins to which the altered glycans bind, and the roles of the glycans in the functional modulation of glycoproteins in malignant breast cancer cell-CM-treated MCF10A cells.

## 4. Materials and Methods

### 4.1. Cell Lines and Cell Culture

Immortalized human mammary epithelial cell line MCF10A and human breast cancer cell lines MDA-MB-231 and MDA-MB-453 were obtained from American Type Culture Collection (Manassas, VA, USA). The MCF10A cells were cultured in a DMEM/F12 medium containing EGF (20 ng/mL), hydrocortisone (0.5 mg/mL), cholera toxin (100 ng/mL) (Sigma-Aldrich; St. Louis, MO, USA), insulin (10 μg/mL), and 1× penicillin-streptomycin (Gibco; Carlsbad, CA, USA). The human breast cancer cells were cultured in DMEM (HyClone; Logan, UT, USA) supplemented with 10% fetal bovine serum (FBS; Gibco) and 1× penicillin-streptomycin. All cultures were maintained at 37 °C in a 5% CO_2_ atmosphere.

### 4.2. Antibodies and Reagents

The antibodies used were mouse anti-E-cad IgG2a (BD Biosciences; San Jose, CA, USA), mouse anti-*N*-cad IgG1 and anti-TWIST1 (Santa Cruz Biotechnology; Santa Cruz, CA, USA), mouse anti-vimentin IgG1, rabbit anti-FN IgG and anti-GAPDH (Sigma-Aldrich), horseradish peroxidase (HRP)-labeled goat anti-mouse IgG, and HRP-labeled goat anti-rabbit IgG (Beyotime Institute of Biotechnology; Haimen, China). TGF-β was used, with 2 ng/mL as the final concentration (BD Bioscience). Other reagents were from Sigma-Aldrich unless described otherwise.

### 4.3. Conditioned Medium (CM)

Human breast cancer cells were cultured as above in 9 cm plates to confluency, rinsed with 1× PBS, then cultured in DMEM with 1% FBS. CM (termed “231-CM” or “453-CM”) from these cells was harvested after 2 days, filtered through a Stericup (pore size 0.45 μm; Millipore; Billerica, MA, USA) to remove cells and debris, aliquoted, and stored at −80 °C for later experiments.

### 4.4. Total Protein Extraction

The MCF10A cells were grown for 24 h in a culture medium containing 10% FBS, and the medium was then supplemented with TGF-β or CM. The cells were lysed with a T-PER Tissue Protein Extraction Reagent (Thermo Scientific; San Jose, CA, USA) as per the manufacturer’s instructions. In brief, cell pellets were washed twice with ice-cold 1× PBS and lysed with T-PER containing protease inhibitors (0.1% aprotinin, 1% phenylmethanesulfonyl fluoride, and 1% phosphatase inhibitor cocktail) for 30 min on ice, the solution was centrifuged at 12,000 rpm for 15 min (4 °C), and the supernatant was collected and stored at −80 °C. The protein concentration was determined using a BCA kit (Beyotime). Protein lysates were stored for subsequent Western blot analysis, lectin microarray analysis, or *N*-glycan purification.

### 4.5. Proliferation (MTT) Assay

Cell proliferation was determined by MTT assay as described previously [36]. In brief, cells (4 × 10^3^/well) were seeded in 96-well plates and treated with TGF-β or CM. After 24, 48, or 72 h of culture, each well was added with 10 μL MTT solution (Sangon Biotech; Shanghai, China) and incubated for 4 h at 37 °C. The reaction was stopped by an addition of 150 μL DMSO (dimethylsulphoxide), and the absorbance at 490 nm was recorded immediately.

### 4.6. Wound Assay

The wound assay was performed as described previously [37]. Cells (2 × 10^6^/well) were cultured in 6-well plates overnight. In each well, three separate wounds were scratched using a 200-μL pipette tip. The cells were rinsed twice with fresh serum-free medium, cultured in DMEM with 1% FBS, and then treated with TGF-β or CM. After 0, 24, or 48 h of incubation, the wounds at the marked lines were photographed, and the areas occupied by moving cells were calculated.

### 4.7. Apoptosis Detection

Apotosis detection was performed as described in the PE Annexin V Apoptosis Detection Kit I (BD Pharmingen; San Jose, CA, USA). In brief, 2 × 10^5^ cells were cultured in 6-well plates overnight and treated with TGF-β or CM for 24 h. Then, the cells were washed twice with cold PBS and resuspended in binding buffer at a concentration of 10^6^ cells/mL. Further, 5 μL of PE Annexin V or 5 μL of 7-AAD or both were added, and incubated for 15 min at room temperature in the dark. Finally, 400 μL binding buffer was added to each tube and detected by BD Accuri C6 flow cytometry (BD Biosciences) through Channel FL2 and FL3. The results were analyzed by FlowJo VX (Tree Star Inc., Ashland, OR, USA). All of the experiments were performed three times.

### 4.8. Release and Purification of N-Glycans

Proteins were concentrated and desalted using a size-exclusion spin ultrafiltration unit (Amicon Ultra-0.5 10 KD device; Millipore) [38] and denatured with 8 M urea, 10 mM DTT (dithiothreitol), and 10 mM IAM (iodoacetamide). Denatured proteins were removed, and the samples in the unit were further incubated overnight with PNGase F (New England BioLabs; Ipswich, MA, USA) at 37 °C to release *N*-glycans. The *N*-glycans were eluted with Milli-Q water and desalted as described previously [38], dissolved in 1-butanol/methanol/H_2_O (5:1:1) (BMW), and added to Sepharose 4B, which was then equilibrated with methanol/H_2_O (1:1) (MW) and BMW. After incubation for 45 min, the *N*-glycans were washed with BMW, eluted with MW, and lyophilized.

### 4.9. MALDI-TOF/TOF-MS Analysis of N-Glycans

*N*-glycans were dissolved in 10 μL MW and spotted on an MTP AnchorChip (Bruker Daltonics; Bremen, Germany) sample target. The samples were air-dried, and 1 μL DHB solvent (20 mg/ mL in MW) was added to recrystallize the glycans. The *N*-glycans were analyzed by MALDI-TOF/TOF-MS (UltrafleXtreme; Bruker Daltonics) in positive ion mode, and *m*/*z* data were analyzed and annotated using the GlycoWorkbench software program as described previously [7]. The relative variation of the different types of *N*-glycans were calculated by adding up all of the relative intensity of a given type of *N*-glycan.

### 4.10. Lectin Microarray Analysis and Data Analysis

The lectin microarray analysis and data analysis were performed as described previously [39,40]. In brief, lectin microarrays were produced by spotting 37 lectins obtained from Vector Laboratories (Burlingame, CA, USA), Sigma-Aldrich, and Calbiochem Merck (Darmstadt, Germany) onto epoxysilane-coated slides. Glycoprotein samples labeled with fluorescent dye Cy3 (GE Healthcare; Buckinghamshire, UK) were applied to the microarrays, which were then scanned with a confocal scanner (model GenePix 4000B; Axon Instruments; Union City, CA, USA). The normalized data for a given experimental group and corresponding control group were compared to determine the relative change in protein glycosylation.

### 4.11. Lectin Staining

MCF10A cells (2 × 10^4^/well in a 24-well plate) were cultured on sterile coverslips and treated with 231-CM for 48 h. The cells were: (i) fixed with 2% paraformaldehyde for 15 min and permeabilized with 0.2% Triton X-100 in PBS for 10 min; (ii) washed twice with PBS and blocked overnight with 5% (*w*/*v*) BSA in PBS at 4 °C; (iii) incubated with Cy3-labeled lectin (15-20 μg/ mL) in 5% BSA for 3 h in the dark, and stained with DAPI (20 μg/ mL) in PBS for 10 min; (iv) rinsed with PBS to remove extra dye; and (v) mounted with Glycergel (DakoCytomation; Carpinteria, CA, USA) and observed by fluorescence microscopy (model Eclipse E600; Nikon; Tokyo, Japan).

### 4.12. Statistical Analysis

The data were analyzed by a two-tailed, two-sample t-test with assumption of equal variance, using the GraphPad Prism 5 software program (GraphPad Software; La Jolla, CA, USA). Differences between means were considered statistically significant at *p* ≤ 0.05.

## Figures and Tables

**Table 1:** Proposed structures and their molecular ions in MALDI-TOF/TOF-MS spectra of *N*-glycans from the control and 231-CM-treated MCF10A cells.

Experimental Spectrum		Glycan Structure	Relative Intensity	
*m*/*z*	Type		MCF10A	MCF10A + 231-CM
1406.633	M + Na^+^	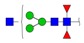	0.0497 ± 0.0016	0.0502 ± 0.0022
1419.414	M + Na^+^	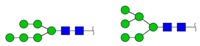	0.0816 ± 0.0066	0.0464 ± 0.0054
1581.461	M + Na^+^	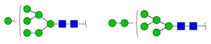	0.0930 ± 0.0052	0.0601 ± 0.0011
1590.471	M + Na^+^	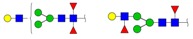		0.0207 ± 0.0002
1641.893	M + Na^+^	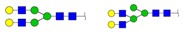	0.0278 ± 0.0060	0.0326 ± 0.0095
1668.782	M + Na^+^	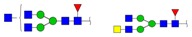		0.0266 ± 0.0010
1743.519	M + Na^+^	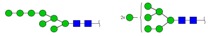	0.1904 ± 0.0026	0.2184 ± 0.0448
1905.570	M + Na^+^	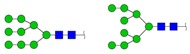	0.0821 ± 0.0047	0.0993 ± 0.0097
2291.107	M + Na^+^	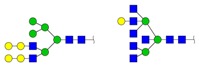	0.0188 ± 0.0050	0.0153 ± 0.0012
2421.141	M + Na^+^	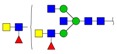	0.0192 ± 0.0009	0.0286 ± 0.0057
2677.226	M + Ah (Acethydrazide) + Na^+^	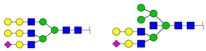		0.0170 ± 0.0036
2824.216	M + Ah + Na^+^	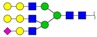	0.0505 ± 0.0022	0.0818 ± 0.0117
2841.237	M + Ah + Na^+^	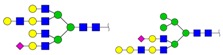		0.0114 ± 0.0015
2988.342	M + Na^+^	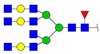	0.0113 ± 0.0000	
3082.257	M + Ah + Na^+^	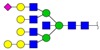		0.0102 ± 0.0000
3228.304	M + Na^+^M + Ah + Na^+^M + 2Ah + Na^+^	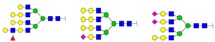	0.0199 ± 0.0000	0.0116 ± 0.0010
3399.292	M + Na^+^M + Ah + Na^+^M + 2Ah + Na^+^	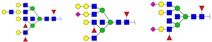	0.0179 ± 0.0000	0.0143 ± 0.0010

**Table 2:** Relative variation of various types of *N*-glycans in DMEM/1% FBS-incubated and 231-CM-treated MCF10A cells.

Glycan Types	Relative Variation (%)	
	MCF10A	MCF10A + 231-CM
Hybrid	10.35	13.09
High-mannose	67.50	56.99
Complex	22.14	29.92
Biantennary	8.69	10.89
Triantennary	9.22	10.55
Tetra-antennary	18.97	40.56
Bisecting GlcNAc	16.32	45.46
Fucosylated	17.71	45.91

**Table 3:** Lectin microarray analysis of glycans showing significantly different expression in DMEM/1% FBS-incubated vs. 231-CM-treated MCF10A cells.

Lectin	Abbreviation	Specificity	Fold Change
Upregulated			
*Bandeiraea simplicifolia*	BS-I	α-Gal (galactose) and α-GalNAc (*N*-acetylgalactosamine)	25.7155
*Lycopersicon esculentum* (tomato) lectin	LEL	sialylated and terminal Gal/GalNAc structures	5.1599
*Solanum tuberosum* (potato) lectin	STL	GlcNAc oligomer	4.1605
*Psophocarpus tetragonolobus* lectin I	PTL-I	αGalNAc and Gal	3.8853
*Psophocarpus tetragonolobus* lectin II	PTL-II	Gal	2.9439
*Sambucus nigra* lectin	SNA	Sia2-6Galβ1-4Glc(NAc)	2.6970
*Maclura pomifera* lectin	MPL	αGalNAc	2.0992
Downregulated			
*Phaseolus vulgaris* agglutinin(E)	PHA-E	Bisecting GlcNAc and biantennary *N*-glycans	0.6670
*Wisteria floribunda* lectin	WFA	Terminal GalNAc	0.5197
*Griffonia (Bandeiraea) simplicifolia* lectin I	GSL-I	αGalNAc, GalNAcα-Ser/Thr (Tn), and αGal	0.5126
*Canavalia ensiformis*	ConA	branched and terminal mannose, terminal GlcNAc	0.4926
*Sophora japonica* agglutinin	SJA	Terminal GalNAc and Gal	0.3750
*Peanut* agglutinin	PNA	Galβ1-3GalNAcα-Ser/Thr(T)	0.3664
*Ulex europaeus* agglutinin I	UEA-I	Fucα1-2Galβ1-4Glc(NAc)	0.2769
*Ricinus communis* agglutinin I	RCA120	β-Gal	0.2070
*Phaseolus vulgaris* agglutinin(E + L)	PHA-E+L	Bisecting GlcNAc, biantennary *N*-glycans, and tetra-antennary complex-type *N*-glycan	0.1534
*Aleuria aurantia* lectin	AAL	Fuc	0.1521
*Bauhinia purpurea* lectin	BPL	Galβ1-3GalNAc	0.0939
*Soybean* agglutinin	SBA	Terminal GalNAc (particularly GalNAcα1-3Gal)	0.0703
*Datura stramonium*	DSA	GlcNAc	0.0106
*Artocarpus integrifolia*	jacalin	Galβ1-3GalNAcα-Ser/Thr(T)and GalNAcα- Ser/Thr(T)	0.0072
*Erythrina crista-galli*	ECA	Galβ-1,4GlcNAc	0.0017
*Euonymus europaeus* lectin	EEL	Galα1-3(Fucα1-2)Gal	0.0006

## Supplementary Materials

Supplementary materials can be found at www.mdpi.com/1422-0067/18/8/1528/s1.
