# Supplementary material for: Conditioned Medium from Malignant Breast Cancer Cells Induces an EMT-Like Phenotype and an Altered N-Glycan Profile in Normal Epithelial MCF10A Cells
Source: Int J Mol Sci. 2017 Aug 1;18(8):1528. doi: 10.3390/ijms18081528 (PMC5577993; doi:10.3390/ijms18081528)
Supplement: Supplementary file 1 [file ijms-18-01528-s001.pdf]

Table S1. Proposed structures and their molecular ions in MALDI-TOF/TOF-MS spectra of N-glycans from control and 453-CM-treated MCF10A cells.

No.	Experimental *m/z*	Calculated *m/z*	Type	Glycan Structure	Relative Intensity	
					MCF-10A	MCF-10A+453-CM
1	1135.4716	1136.3964	M+Na$^+$	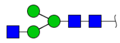	0.007	ND
2	1257.52	1257.4226	M+Na$^+$	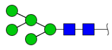	0.023	0.014
3	1406.928	1406.663	M+Na$^+$	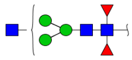	0.010	0.011
4	1419.584	1419.4775	M+Na$^+$	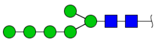	0.082	0.088
5	1581.652	1581.5283	M+Na$^+$	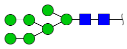	0.132	0.129
6	1590.271	1590.471	M+Na$^+$	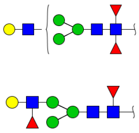	ND	0.005
7	1668.592	1668.782	M+Na$^+$	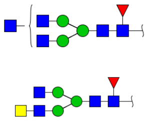	0.013	0.015
8	1743.722	1743.5881	M+Na$^+$	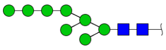	0.333	0.340
9	1865.7683	1867.6571	M+H$^+$	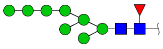	0.011	0.008
10	1905.796	1905.6339	M+Na$^+$	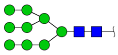	0.198	0.185
11	1955.802	1955.6972	M+Na$^+$	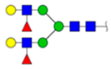	0.015	0.017
12	1969.877	1971.6921	M+Na$^+$	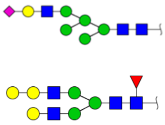	ND	0.006
13	2010.901	2012.7187	M+Na$^+$	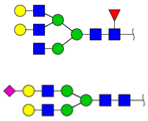	0.014	0.014
14	2065.901	2067.6868	M+Na$^+$	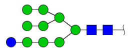	0.010	0.009
15	2067.876	2069.7401	M+Na$^+$	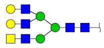	0.012	ND
16	2101.8652	2101.7551	M+Na$^+$	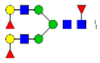	0.011	0.011
17	2117.8689	2117.75	M+Na$^+$	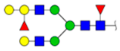	0.008	0.010
18	2156.966	2158.7766	M+Na$^+$	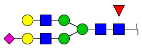	0.015	0.020

				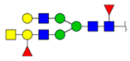		
19	2174.972	2174.7715	M+Na+	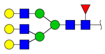	0.009	0.009
20	2212.9369	2213.7447	M+Na+	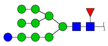	0.008	0.007
21	2263.94	2263.8079	M+Na+	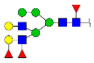	0.007	0.008
22	2302.9582	2304.8345	M+Na+	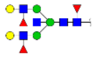	0.009	0.011
23	2319.9697	2320.8294	M+Na+	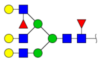	0.007	0.006
24	2358.027	2358.027	M+Na+	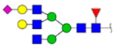	0.019	0.021
25	2421.368	2421.141	M+Na+	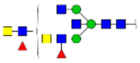	0.004	0.008
26	2504.0870	2504.0870	M+Na+	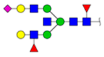	0.005	0.006
27	2522.0800	2522.0800	M+Na+	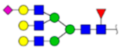	ND	0.003
28	2560.106	2558.9483	M+H+	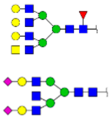	ND	0.004
29	2723.133	2667.133	M+Na+	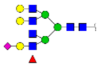	0.004	0.003
30	2869.2300	2813.2300	M+Na+	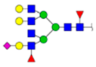	ND	0.003
31	2988.103	2988.342	M+Na+	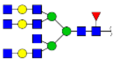	0.004	0.003
32	3068.1506	3067.0887	M+Na+	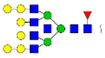	0.010	ND
33	3070.33	3070.33	M+Na+	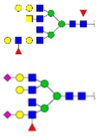	0.011	0.019
34	3124.563	3012.563	M+H+	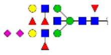	0.004	0.009
35	3126.275	3070.275	M+H+	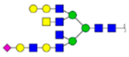	0.009	ND

Table S2. Relative variation of various types of *N*-glycans in DMEM/ 1% FBS-incubated and 453-CM-treated MCF10A.

**Glycan Types**	**Relative variation (%)**	
	MCF10A	MCF10A+453-CM
Hybrid	0.7	1.4
High-mannose	79.6	77.9
Complex	20.5	21.2
Biantennary	7.7	9.5
Tri- and Tetra-antennary	10.4	10.2
Fucosylation	17.0	21.3
Bisecting GlcNAc	3.5	3.7
